# Population-Based Incidence of Typhoid Fever in an Urban Informal Settlement and a Rural Area in Kenya: Implications for Typhoid Vaccine Use in Africa

**DOI:** 10.1371/journal.pone.0029119

**Published:** 2012-01-19

**Authors:** Robert F. Breiman, Leonard Cosmas, Henry Njuguna, Allan Audi, Beatrice Olack, John B. Ochieng, Newton Wamola, Godfrey M. Bigogo, George Awiti, Collins W. Tabu, Heather Burke, John Williamson, Joseph O. Oundo, Eric D. Mintz, Daniel R. Feikin

**Affiliations:** 1 Global Disease Detection Division, Kenya Office of the US Centers for Disease Control and Prevention, Nairobi and Kisumu, Kenya; 2 Kenya Medical Research Institute (KEMRI), Nairobi and Kisumu, Kenya; 3 Kenya Ministry of Public Health and Sanitation, Nairobi, Kenya; 4 National Center for Emerging and Zoonotic Infectious Diseases, Centers for Disease Control and Prevention, Atlanta, Georgia, United States of America; London School of Hygiene and Tropical Medicine, United Kingdom

## Abstract

**Background:**

High rates of typhoid fever in children in urban settings in Asia have led to focus on childhood immunization in Asian cities, but not in Africa, where data, mostly from rural areas, have shown low disease incidence. We set out to compare incidence of typhoid fever in a densely populated urban slum and a rural community in Kenya, hypothesizing higher rates in the urban area, given crowding and suboptimal access to safe water, sanitation and hygiene.

**Methods:**

During 2007-9, we conducted population-based surveillance in Kibera, an urban informal settlement in Nairobi, and in Lwak, a rural area in western Kenya. Participants had free access to study clinics; field workers visited their homes biweekly to collect information about acute illnesses. In clinic, blood cultures were processed from patients with fever or pneumonia. Crude and adjusted incidence rates were calculated.

**Results:**

In the urban site, the overall crude incidence of *Salmonella enterica* serovar Typhi (*S*. Typhi) bacteremia was 247 cases per 100,000 person-years of observation (pyo) with highest rates in children 5–9 years old (596 per 100,000 pyo) and 2–4 years old (521 per 100,000 pyo). Crude overall incidence in Lwak was 29 cases per 100,000 pyo with low rates in children 2–4 and 5–9 years old (28 and 18 cases per 100,000 pyo, respectively). Adjusted incidence rates were highest in 2–4 year old urban children (2,243 per 100,000 pyo) which were >15-fold higher than rates in the rural site for the same age group. Nearly 75% of *S*. Typhi isolates were multi-drug resistant.

**Conclusions:**

This systematic urban slum and rural comparison showed dramatically higher typhoid incidence among urban children <10 years old with rates similar to those from Asian urban slums. The findings have potential policy implications for use of typhoid vaccines in increasingly urban Africa.

## Introduction

With improvements in municipal drinking water treatment, sanitation, hygiene, and food production and preparation, illness and death from typhoid fever, once rampant in New York, London and other Western cities in the late 1800′s 1–3, became rare in industrialized nations during the 20^th^ Century [2]–[4]. By contrast, well into the 21^st^ Century, typhoid remains a problem in lesser developed countries [5]. Most recent focus on typhoid disease burden has been on Asian urban centers, where high incidence rates have been documented within urban slums [5], [6]. However, little attention has been paid to typhoid prevention in Africa, where there have been few systematic investigations, and where a recent study from a rural area showed low incidence [7].

Since 2006, we have collected population-based surveillance data for infectious disease syndromes in an urban informal settlement and from a rural area in Kenya to provide data for use in characterizing emerging pathogens, estimating disease burden, defining priorities for public health research and interventions, and for sites to evaluate the impact of new interventions [8], [9]. We set out to compare incidence of *Salmonella enterica* serovar Typhi (*S*. Typhi) bacteremia in a densely populated urban slum and a rural community in Kenya, hypothesizing higher rates in the urban area, given crowding and suboptimal access to safe water, sanitation and hygiene and the high rates within urban settings in Asia with similar characteristics.

## Methods

**Figure 1:**
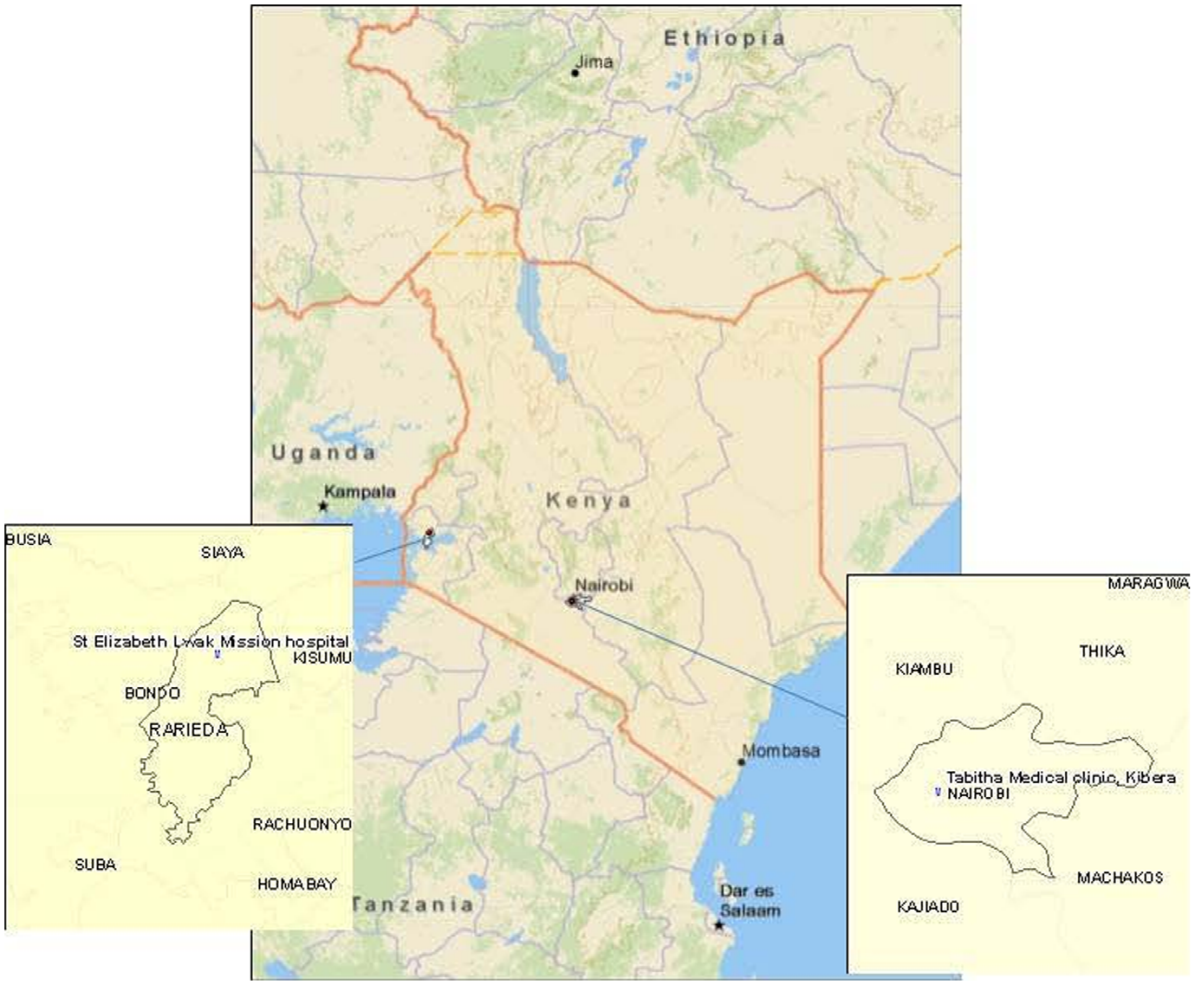
Map of Kenya with Insets Showing Location of Study Sites.

The Kenya Medical Research Institute-Centers for Disease Control and Prevention collaboration (KEMRI-CDC) has conducted active population-based surveillance for febrile illness, pneumonia, diarrheal disease and jaundice within two of 12 neighborhoods or “villages” in Kibera, Nairobi, Kenya and in Lwak in rural western Kenya within a district currently known as Rarieda in Nyanza Province, since October 2005 (Figure 1). The Kibera surveillance area is 0.40 km^2^ and includes approximately 28,000 participants of all ages (71,000 people/km^2^). In Lwak the surveillance area is 100 km^2^ and there are approximately 25,000 participants of all ages (325 people/km^2^). Consenting households (<1% of heads of households refused to participate) were visited every 2 weeks by community interviewers who collected standardized information into pre-programmed personal data assistants (PDA) about illnesses in residents and healthcare-seeking associated with that illness, as described in detail previously [8], [9]. An existing clinic, known as the Tabitha Clinic, operated by Carolina for Kibera, was enhanced to serve as the Kibera site field clinic, and 6 clinicians (medical officers and clinical officers) and 7 nurses were hired and trained on standard medical practices, including treatment algorithms and on surveillance and diagnostic criteria according to the study protocol. We also hired a clinical officer and 2 nurses to work with existing health care providers at Lwak Mission Hospital which serves as the Lwak field clinic. Specimens tested for this study were only collected at the field clinics; no specimens were collected during home visits.

The clinical component of the surveillance system has been described [9]. Briefly, participants were allowed free access to the two study clinics, Tabitha (Kibera) and Lwak Mission Hospital (Lwak), for any acute medical condition, were clinically assessed, and provided with medications and follow-up without charge; data were systematically collected for all patients at both sites [9]. During home visits, community interviewers encouraged ill participants to visit the field clinic; however, no financial incentives, travel reimbursements, or direct transport were provided. Clinicians used their clinical judgment when deciding whether to treat empirically with an antimicrobial drug before blood culture results were available. Study team members went to homes of patients from whom S. Typhi was isolated from blood culture and asked them to return to clinic where empirical therapy (before drug susceptibility testing was complete) generally included either ciprofloxacin or ceftriaxone; very ill patients were referred to Mbagathi Hospital.

**Figure 2.**
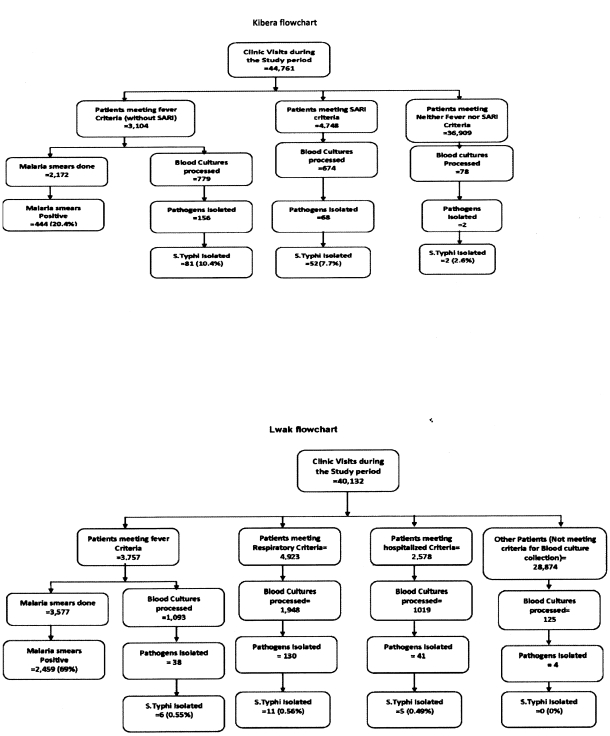
a) Flow Chart showing Numbers of Enrollees from Lwak, Blood Cultures Processed and Pathogens Isolated, Including *S* Typhi, Isolated. b) Flow Chart showing Numbers of Enrollees from Kibera, Blood Cultures Processed and Pathogens Isolated, Including *S* Typhi, Isolated.

Blood cultures (one per patient) were obtained from patients who met respiratory and febrile illness case definitions (see below) (Figures 2a and 2b). Malaria smears were collected at the discretion of clinicians. Also, in Lwak, all patients who were hospitalized (except for reasons that were obviously not related to infection, like trauma) underwent blood culture before being given antimicrobial drugs. In some cases, clinicians in both sites ordered a blood culture from patients who did not meet fever or respiratory criteria if they had clinical suspicion of sepsis. Written informed consent was collected for all participants involved in this study. In Kibera, febrile illness was defined as a documented axillary temperature of ≥38.5^o^C until November 1, 2008, when the threshold was decreased to ≥38.0^o^C. For the entire study period in Lwak, febrile illness was defined as a temperature ≥38.0^o^C. In Kibera, all consenting patients with febrile illness were offered blood culture. In Lwak, because of a higher expected incidence of febrile illness due to malaria, blood cultures were collected each day from only the first two persons≥5 years old and the first two children <5 years old. Respiratory illness was defined for children <5 years old as: cough OR difficulty breathing AND one of the following: convulsions, unable to drink fluids or unable to breastfeed, lethargic, chest indrawing, vomiting everything, stridor, oxygen saturation <90%; and for persons ≥5 years old as cough OR difficulty breathing OR chest pain AND one of the following: temperature ≥38.0^o^C and oxygen saturation <90%.

For children ≤5 years of age, 1–3 mL of blood was collected and placed into a BACTEC Ped Plus/F (enriched Soybean-casein digest broth with CO_2_; Becton-Dickenson) blood culture bottle. For participants >5 years old, we attempted to collect 8–10 mL of blood, which was placed into a BACTEC Plus/F (for adults) bottle. We processed blood cultures when amount of blood collected was less than the desired volume (majority of specimens); however, isolation rates did not vary by volume of blood cultured (data not shown).

Blood culture bottles from Kibera were incubated in a Bactec 9050 system at the KEMRI-CDC laboratory in Nairobi. Bottles signaling positive from Kibera were sent by overnight mail to the KEMRI-CDC laboratory in Kisumu. Bottles collected in Lwak were also placed in a BACTEC 9050 system at the Kisumu KEMRI-CDC laboratory. Subcultures were processed using standard microbiological procedures [10]. *Salmonella* isolates were identified and confirmed using an API 20E system (Appareils et Procedes d'Identification, Montalieu Vercieu, France) following manufacturers' instructions. Commercial agglutinating antiserum (Denka Seiken, Tokyo, Japan) was used to serotype the *Salmonella* isolates [10]. Antimicrobial susceptibility testing was performed on *Salmonella* isolates by Kirby-Bauer disc diffusion [11] and for the purposes of this report, interpreted according to the latest Clinical and Laboratory Standards Institute guidelines [12]. Multi-drug resistant *S*. Typhi was defined by resistance to chloramphenicol, ampicillin and trimethoprim [13], [14].

For the home visits, data were collected by trained community interviewers on pre-programmed personal digital assistants (PDA) with drop-down response menus and validation checks programmed in Microsoft Visual Studio.Net 2005. Kibera clinical data were entered into a customized computer-based health care information system, developed by GFL Information, Nairobi, Kenya. In Lwak, clinical data were entered onto teleforms, standardized for this project (with the same fields used as the Kibera computer-based system), and scanned and reviewed by data clerks for accuracy and completeness. Statistical analysis was performed using SAS system for Windows (SAS Institute, Cary, NC) version 9.1.

Blood culture collection and processing began in March 2007 in Kibera and in October 2006 in Lwak. We analyzed data from Kibera for March 1, 2007- February 28, 2009 and from October 1, 2006 to September 30, 2009 for Lwak.

Overall and age-group incidence rates were calculated by dividing number of cases of *S*, Typhi bacteremia by number of person-years of observation (pyo); permanent residence status on enrolled participants in surveillance areas was used to determine start and stop dates for person-time contribution for each participant [9]. Start date was either the date that the study period began for those residents leaving in the surveillance area at the time the study began, or the date at which a new resident (an “in-migrant”) had completed documented residence (as determined by community interviewers) within the study area for a period 4 months (8 biweekly home visits by community interviewers). Stop date was either the end of the study period or the date that a participant moved away from the study area (out-migrant), as determined during home visits by community interviewers. The period between start and stop dates for each resident (in days) was divided by 365.25 to determine pyo, which was then totaled for all participants (or all participants within defined age groups). We calculated age-adjusted incidence rates to account for cases of *S.* Typhi bacteremia missed. We made two age group-specific adjustments. The first adjustment (Adjustment #1) was based on patients who visited the study clinic and did not have a blood culture done, despite meeting fever and respiratory case definitions—this took into account that in Lwak only the first two children <5 and the first two participants ≥5 years old with documented fever underwent blood culture—the adjustment put the number of people who met surveillance criteria for blood culture in the denominator and the number of patients who actually had blood culture done in the numerator; we divided number of participants with febrile illness criteria and ALRI criteria who had blood culture obtained by number of all surveillance participants presenting to study clinic meeting those criteria. The principal reasons in Kibera for not having a blood culture obtained were that phlebotomy was unsuccessful or that patient/caretaker did not want to wait additional time. The resulting proportions were divided into crude incidence rates. The second extrapolation (Adjustment #2) was based on number of patients whose indication for blood culture was either febrile illness or ALRI, based on data from the home visit, who visited a clinic, but not the study clinic, and, thus, did not have the opportunity for blood culture, since no other clinics in either area performed them. We assumed that patients meeting illness criteria who visited another clinic in the area were no different than those that visited the study clinic in likelihood of having typhoid fever. Thus, we divided number of residents (within each age-group category) with history of fever or ALRI on home visit who visited the study clinic by number of residents with history of fever who visited any clinic for that illness. Episodes of fever not associated with visits to any clinic were not “counted” in the adjustment. This proportion was divided into the rate Adjustment #1 figure for each age group; the result represented final age-specific adjusted rates (Adjustment # 2). Exact 95% confidence intervals were calculated for the unadjusted incidence rates except when the number of cases was 0. Ninety-five percent confidence intervals were calculated for the adjusted incidence rates by adjusting the lower and upper ends of the exact 95% confidence intervals for the unadjusted rates accordingly.

The protocol was reviewed and approved by the Ethical Review Committee at the Kenya Medical Research Institute and the Institutional Review Board of CDC-Atlanta.

## Results

*S*. Typhi was isolated from 137 (6.4%) of 2,142 blood cultures processed from Kibera (during the 2-year study period) (Table 1) and from 22 (0.6%) of 3,577 blood cultures from rural Lwak (during the 3-year study period). *S*. Typhi was isolated twice from blood cultures collected from two patients within a 7-day period in Kibera, which we categorized as persistent bacteremia in single cases of typhoid illness, leaving 135 discrete cases of bacteremia in Kibera. The overall contamination rate of blood cultures was 7.4%; *S* Typhi was not isolated from any blood culture from which a contaminant was grown.

**Table 1:** Isolation rates for *S*. Typhi from blood cultures collected in Kibera and Lwak.

Age	*S*. Typhi isolates(Column % by age)	Any pathogen isolated(Row % *S*. Typhi*)	Blood cultures done(Row % *S*.Typhi**)
A. KIBERA			
<2 years	3 (2%)	17 (18%)	353 (0.8%)
2–4 years	32 (24%)	52 (62%)	677 (4.7%)
5–9 years	48 (36%)	71 (68%)	454 (10.6%)
10–17 years	24 (18%)	31 (77%)	189 (12.7%)
18–34 years	25 (19%)	45 (56%)	341 (7.3%)
35–49 years	2 (2%)	37 (5%)	106 (1.9%)
≥50 years	1 (1%)	2 (50%)	22 (4.5%)
Total	135&	225 (60%)	2142 (6.3%)

B. LWAK			
<2 years	1 (5%)	36 (3%)	758 (0.1%)
2–4 years	2 (9%)	33 (6%)	1018 (0.2%)
5–9 years	2 (9%)	20 (10%)	770 (0.3%)
10–17 years	4 (18%)	16 (25%)	402 (1.0%)
18–34 years	11 (50%)	57 (19%)	447 (2.5%)
35–49 years	0 (0%)	26 (0%)	244 (0.0%)
≥50 years	2 (9%)	25 (8%)	196 (1.0%)
Total	22	213 (10%)	3835 (0.6%)

*Proportion of pathogens isolated that were *S*. Typhi by age group.

**Proportion of blood cultures done from which *S*. Typhi was isolated.

& Two patients had *S*. Typhi isolated from blood cultures twice during a seven day period (representing persistent bacteremia); for the purposes of these rate calculations, we excluded one of the blood cultures for each of these patients. We did not have age information for one of the patients.

The highest *S*. Typhi isolation rates (13%) in Kibera were among 10–17 year olds, followed by 5–9 year olds (11%) (Table 1). Of the 135 cases in Kibera, 51 (37.8%) had blood cultures collected because they met the case definition for acute respiratory infection. There were no deaths within 30 days of onset of illness and 2 (1.5%) patients were hospitalized.

In contrast to the urban and rural differences for isolation of *S*. Typhi, non-typhoidal *Salmonella* (*Salmonella* Enteritidis and *Salmonella* Typhimurium) were isolated from a much higher proportion of cultures from Lwak, when compared with Kibera (1.6% versus 0.3%;p<0.0001). *Salmonella* Paratyphi was not isolated from any blood cultures.

The crude incidence of blood culture-confirmed typhoid fever in Kibera was 247 cases per 100,000 pyo compared with 29 cases per 100,000 pyo in Lwak. The crude rates in Kibera were highest in children 5-to-9 and 2-to-4 years old (596 and 521 cases per 100,000 pyo, respectively) (Table 2); by contrast, the highest rates in Lwak were in the 18–34 year age group (63 cases per 100,000 pyo) with very low rates in children 2–4 and 5–9 years old (28 and 18 cases per 100,000 pyo, respectively) (Table 3).

**Table 2:** Crude and Adjusted Rates of S Typhi Bacteremia in Kibera.

Agein years	*Syndrome*	S.typhi(n)	Pyo*	Crude Rate per100,000pyo	% cultured	Rate,Extrapolation 1**(Extrapolated No. of typhi cases)	% clinicvisits toTabitha	Adjusted RateExtrapolation 2***(95% CI)
0–1	**Overall** *SARI Outpatient* *Fever, not SARI*	321	3,457	**86.8**57.928.9	14.021.0	**549.6**(19)405.0(14)144.6(5)	66.9	821.5(265–2547)
2–4	**Overall** *SARI Outpatient* *Fever, not SARI*	32923	6,138	**521.3**146.6374.7	41.035.2	**1,417.3**(87)358.4(22)1,058.9(65)	63.2	2,242.6(1586–3171)
5–9	**Overall** *SARI Outpatient* *Fever, not SARI*	482622	8,049	**596.3**323.0273.3	65.238.4	**1,205.1**(97)496.9(40)708.1(57)	67.4	1,788.0(1348–2373)
10–17	**Overall** *SARI Outpatient* *Fever, not SARI Others*	245181	8,017	**299.4**62.4224.512.5	65.543.83	**623.7**(50)99.8(8)511.4(41)12.5(1)	71.7	869.9(583–1298)
18–34	**Overall** *SARI Outpatient* *Fever, not SARI* *Others*	259151	20,309	**123.1**44.373.94.9	65.157.0	**201.9**(41)68.9(14)128.0(26)4.9(1)	64.7	312.1(211–462)
35–49	**Overall** *SARI Outpatient* *Fever, not SARI*	202	6,443	**31.0**0.0**31.0**	80.047.2	**62.1(4)**0.0(0)62.1(4)	62.1	100.0(25–400)
>50	**Overall** *SARI Outpatient* *Fever, not SARI*	110	2,120	**47.2**47.20.0	73.3100.0	**47.2(1)**47.2(1)0.0(0)	70.9	66.6(13–644)
**>17**	**Overall** *SARI Outpatient* *Fever, not SARI* *Others*	2810171	**28,872**	**97.0**34.658.93.5	56.468.7	**152.4(44)**62.3(18)86.6(25)3.5(1)	**64.5**	231.3(160–335)
**Overall**		**135**	54,535	**247.5**		**548.3(299)**	**66.7**	**822.0** (695–973)

**Table 3:** Crude and Adjusted Rates of S Typhi Bacteremia in Lwak.

Agein years	*Syndrome*	*S*. typhi(n)	Pyo*	Crude Rate per100,000pyo	% cultured	RateExtrapolation 1**(Extrapolated No. of typhi cases)	% clinicvisits toLwak	Adjusted RateExtrapolation 2***(95% CI)
0–1	**Overall** *SARI* *Fever^a^* *Inpatient^b^*	1100	6544.5	15.315.3	19.822.231.7	**76.0(5)**76.0(5)	22.0	345.7 (43–2158)
2–4	**Overall** *SARI* *Fever* *Inpatient*	2110	7027.54	28.514.214.2	24.628.441.4	**156.5 (11)**99.6 (7)56.9(4)	21.1	742.6 (113–1804)
5–9	**Overall** *SARI* *Fever* *Inpatient*	2200	11312.0	17.717.700	49.535.747.6	**35.4(4)**35.4(4)	16.4	215.5 (56–903)
10–17	**Overall** *SARI* *Fever* *Inpatient*	4301	16756.5	23.917.906.0	48.333.341.5	**47.8(8)**35.8(6)011.9(2)	18.3	260.4 (108–767)
18–34	**Overall** *SARI* *Fever* *Inpatient*	11254	17359.5	63.411.528.823.0	63.643.540.6	**144.0(25)**17.3(3)69.1(12)57.6(10)	17.7	815.7(339–1106)
35–49	**Overall** *SARI* *Fever* *Inpatient*	0000	7655.1	**0**	61.250.032.8	0	14.5	0.0
>50	**Overall** *SARI* *Fever* *Inpatient*	2101	10362.0	**19.3**9.709.7	60.138.934.2	**48.3(5)**19.3(2)029.0(3)	8.5	565.8(119–1896)
**>17**	**Overall** *SARI* *Fever* *Inpatient*	13355	35376.6	**37.8**8.714.514.5	62.244.536.3	**84.8(30)**14.1(5)31.1(11)39.6(14)	13.6	625.6(290–860)
**Overall**		**22**	**77017.0**	**28.6**		**75.3(58)**	**16.9**	**445.0 (308–711)**

*pyo – person years of observation.

**Extrapolation 1 accounts for patients meeting case definitions for blood culture in Tabitha clinic who did not have blood cultures done.

*Extrapolation 2 uses the rates for extrapolation 1 and extrapolates further by accounting for the % of persons at the biweekly home visit with SARI or fever for more than 2 days who go to a clinic other than the field clinics:Tabitha (Kibera) or Lwak Mission Hospital (Lwak).

The overall adjusted incidence rate for *S*. Typhi bacteremia in Kibera was 843 cases per 100,000 pyo (Table 2). The adjusted incidence rates in the 2-to-4 and 5-to-9 year age groups in Kibera were 17-fold and 22-fold higher than the adjusted rates for the respective age groups in Lwak (Table 3).

**Figure 3:**
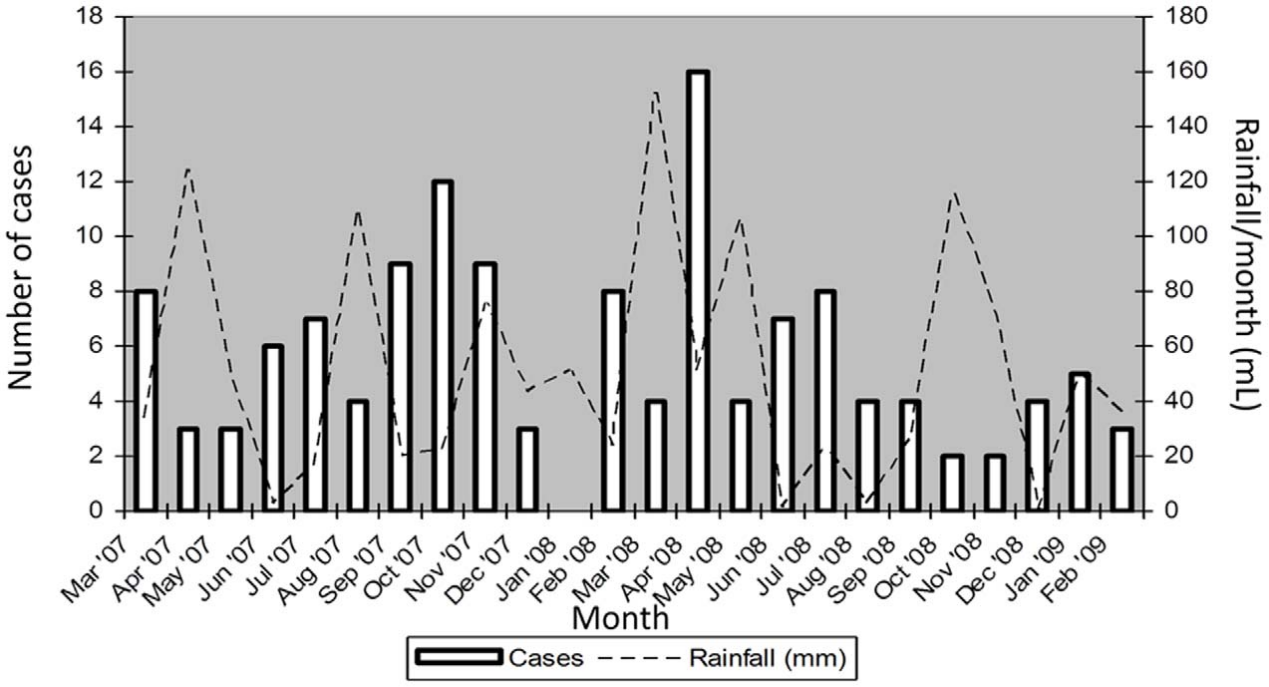
Number of Cases of Typhoid fever and Rainfall Accumulation per Month, Kibera.

Cases occurred throughout the study period in Kibera with no clear seasonality (Figure 3). While rainfall patterns did not correlate (using Pearson correlation coefficient) with disease incidence in general (r = −0.31, p = 0.14), the peak months for cases, April 2008 and October 2007, followed months with peak rainfall by 1-to-2 months (Figure 3). However, other peak rainfall months, like April 2007 and November 2008 were not followed by an increase in cases. Cases in Kibera occurred throughout all 10 pre-determined geographic zones within the study area with zonal incidence rates not differing significantly (data not shown).

Most isolates of *S*. Typhi were resistant to chloramphenicol and ampicillin. (Table 4). While all isolates were susceptible to ciprofloxacin, 7% of strains had decreased susceptibility (3.2% were fully resistant) to nalidixic acid. Nearly all isolates were susceptible to ceftriaxone (Table 4); overall, 77.8% of isolates were multi-drug resistant. The proportions of susceptible isolates for each antimicrobial drug were similar regardless of age group (data not shown).

**Table 4:** *S*. Typhi isolates susceptible to a variety of antimicrobial drugs--Kibera and Lwak.

Drug	Kibera (n = 135) n (%)	Lwak (n = 22)n (%)	Total (n = 157)n (%)
Ampicillin	25 (19.1%)^b^	7 (31.8%)	36 (22.9%)^b^
Ceftriaxone	129 (98.5%)^b^	21 (95.5%)	150 (98.7%)^b^
chloramphenicol	23 (17.4%)^a^	8 (36.4%)	31 (20.1%)^a^
ciprofloxacin	130 (98.5%)^a^	22 (100%)	152 (98.7%)^a^
cotrimoxazole	23 (17.4%)^a^	7 (31.8%)	30 (19.5%)^a^
naladixic acid	123 (93.2%)^a^	21 (95.5%)	144 (93.5%)^a^

a: test not done for 3 isolates.

b: tests not done for 4 isolates.

Self-reported or proxy information from the biweekly home visits in Kibera and Lwak was available for 79 (56%) of 141 cases with fever reported. Duration of fever ranged from 2–36 days (mean =  10.4 days; 95% CI = 8.7–12 days; median = 8 days).

Based on data collected during study clinic visit for 116 patients with *S*. Typhi bacteremia seen at Kibera or Lwak, a variety of symptoms, especially fever (89%), cough (49%), headache (29%), diarrhea (21%) and chills (20%), were more likely to be seen in patients with *S*. Typhi bacteremia than in patients from whom *S.* Typhi was not isolated; however, abdominal pain was the only symptom differentiating patients with *S*. Typhi bacteremia from other bacteremic patients. (Table 5).

**Table 5:** Symptoms from data collected from clinic visit for 116 patients with *S.* Typhi bacteremia from Kibera and Lwak (from whom clinical data were available) compared with other patients with blood cultures processed.

Sign/ Symptom	Confirmed typhoid cases		Patients with bacteremia with pathogens other than *S.* Typhi		Patients without *S. Typhi* isolated from blood culture	
	#	% (n = 116)	#	% (n = 43)	#	%(n = 2,005)
Diarrhea	24	20.7*	8	18.6	212	10.6
Bloody stool	3	2.6***	0	0.0	15	0.7
Documented fever >38C	103	88.8*	35	81.4	1050	52.4
Abdominal pain	23	19.8*,&	2	4.7	114	5.7
Vomiting	26	22.4**	9	20.9	275	13.7
Diff breathing	6	5.2	3	7.0	137	6.8
Cough	57	49.1	28	65.1	863	43.0
Myalgias	11	9.5***	3	7.0	105	5.2
Chills	23	19.8*	5	11.6	181	9.0
Headache	34	29.3*	12	27.9	325	16.2
Runny Nose	41	35.3	20	46.5	642	32.0

*p<0.001 when compared all patients without *S.* Typhi isolated from blood culture.

**p<0.01 when compared all patients without *S.* Typhi isolated from blood culture.

***p<0.05 when compared all patients without *S.* Typhi isolated from blood culture.

& p <0.05 when compared with patients with bacteremia due to pathogens other than *S*. Typhi.

## Discussion

Our findings, the first population-based study from an urban slum in Africa, suggested very high rates of bacteremic typhoid fever. The Kibera crude rates are similar to those from urban slums in several Asian settings, including Kolkata, Jakarta, Karachi and Dhaka, where rates per 100,000 children per year in children 2–4 years of age were 340, 149, 573, and 1,870, respectively [15], [16]. It is likely that because of dense population and severely limited options for sanitation and safe water, people living within urban slums in Africa and in Asia, are at higher risk for typhoid fever. The findings suggest that prevention efforts, including immunization programs, should, at minimum, target impoverished urban slums.

Typhoid fever is estimated to occur annually in >20 million people worldwide [5]. With 1–4% mortality from invasive *S*. Typhi infection, the annual number of deaths is estimated to be 200,000-to-800,000 globally [5], [17]. Global estimates have assumed that most of the disease burden is in Asia [5], but only three vaccine trials and no population-based studies from Africa were available for inclusion in the estimates. In recommending use of typhoid vaccine in school age or preschool children in geographic areas of known high incidence, especially urban centers in Asia, WHO called for more epidemiologic data from Africa to inform recommendations for that continent [6]. Our findings suggest very high incidence in a densely populated urban slum and low incidence in a rural area. In addition, three population-based studies from rural sites in Africa have reported exceedingly low incidence of typhoid fever [7], [18], [19]; however, there are clearly complexities in the epidemiology beyond current understanding. *S*. Typhi can be an important pathogen in rural or peri-urban areas, as shown recently in hospital-based studies in Tanzania, Ghana [20]–[22], and during recent outbreaks of typhoid fever in rural areas in Malawi and Uganda [23], [24]. The differences we observed between the rural and urban sites were not likely due to differences in HIV seroprevalence rates (16–18% in both areas; CDC unpublished data).

In the 1980′s, high rates (661 cases per 100,000 children per year) of bacteremic typhoid fever were suggested in children, 5-to-16 years old, extrapolating incidence data from two unvaccinated groups (representing vaccine trial and non-vaccine trial participants under blood culture surveillance) during a typhoid vaccine trial in a peri-urban township in the Eastern Tranvaal of South Africa [25]. A hospital-based study from urban Kigali, Rwanda during the same decade showed that *S*. Typhi was by far the most commonly isolated pathogen [5.2%] from blood cultures of 900 consecutive febrile children [26]. By contrast, a hospital-based study conducted in the late 1990′s in Blantyre, Malawi found very few *S*. Typhi bacteremias in children, while reporting frequent isolation of non-typhoidal salmonellae in a malaria endemic area; however, the living conditions for patients residing within the hospital catchment area were not described [27].

The urban population in sub-Saharan Africa is expected to double to nearly 800 million people by 2030 [28]. Infrastructure has not kept pace with massive urban in-migration, resulting in expansion and creation of urban slums, creating a variety of public health challenges and the potential for catastrophic epidemics [29], and increasing rates of diseases linked to water, sanitation and hygiene infrastructure, like typhoid fever.

In contrast with earlier data from Karachi suggesting that school-age children are at much higher risk than children <5 years of age [30], our data are consistent with recent data from Asian urban settings where incidence of typhoid fever in younger (pre-school) children (2-to-4 years old) is on par with that for children 5-to- 9 years old [15], [16].

If validated in other urban settings in Africa, the findings from our study would support typhoid immunization of young children with a geographically and environmentally targeted approach for immunization programs, representing a departure from universal immunization strategies that tend to immunize all children in a region regardless of specific location of residence. During the past two decades, new vaccines have become available to prevent high impact illnesses like *Haemophilus influenzae* B disease, pneumococcal disease, hepatitis B, meningococcal meningitis, and rotavirus diarrhea. During the same period, two vaccines for typhoid fever (oral Ty21a, licensed in 1983 and parenterally administered Vi polysaccharide vaccine, licensed in 1994) have been available, but are used almost exclusively by persons from wealthy countries, including military personnel, who travel to the developing world.

Newer typhoid vaccines, including conjugated Vi polysaccharide vaccines and single-dose liquid oral formulations, still in development phase [31]–[33], would be attractive because they could be given to infants as part of routine infant immunization programs and protect children through high incidence ages. As with what transpired in industrialized urban centers at the turn of the twentieth century, dramatic improvements in provision of safe water and sanitation would likely substantially diminish the burden of diseases like typhoid, especially in Kibera and similar settings. Typhoid immunization program implementation would not be straightforward since current vaccines could not be given as part of the routine infant immunization program.

High vaccination rates would likely dramatically reduce the incidence of typhoid fever in urban slum environments, especially in combination with safer water, sanitation and hygiene. Ultimately, the impact on incidence of significant febrile and respiratory illness, hospitalizations and death of a typhoid immunization program could best be evaluated in large demonstration “vaccine probe” projects where interventions are selectively applied in cluster-randomized or sectioned urban slum areas with surveillance for public health outcomes in place within intervention and non-intervention areas.

We found that circulating *S.* Typhi strains were resistant to commonly used antimicrobial drugs, and that 7% of strains had decreased susceptibility to nalidixic acid, suggesting that high levels of fluoroquinolone resistance may soon follow, as has occurred in India and elsewhere [14], [34]. Emergence of ciprofloxacin-resistant of *S*. Typhi will likely increase illness duration, proportion of treatment failures, and cost of treatment, adding to burden of typhoid fever, further highlighting the urgency of implementing effective typhoid immunization programs in targeted settings [5].

A key limitation for this study was that we found very few cases of severe typhoid illness . While intensive surveillance enabled early detection of cases and provided reliable data for adjusting rates, severe disease and poor outcomes were likely averted because study clinics provided high standards of care in areas where health care options are typically limited [35]. Furthermore, the relatively small population limited ability to detect severe disease and death, which tend to occur in a minority of cases. Our active surveillance methods may have also detected self-limited disease, of relatively low public health consequence (though such patients would likely be able to transmit disease to other susceptible people). While the crude rates were as high as those reported from urban sites in Asia, we relied on our active household surveillance to provide one of two adjustment factors. The adjustments depend upon similarities in clinical severity for those who were cultured versus those who were not, which we assumed, but cannot prove. We did not adjust for limited sensitivity of blood cultures (between 30–70%, depending upon volume of blood cultured and presence of antibiotics) [36]–[38], or for the rate of contamination of blood cultures of 7.4%, which as a consequence underestimated the burden of disease. Some of these weaknesses could be addressed partially through hospital-based surveillance in parallel with community/population-based data [35] and via a vaccine probe study, as mentioned above.

Using adjusted incidence rates and recent census data [39], we estimate just over 100,000 cases of typhoid fever per year from urban slums in Kenya. Among children 2–9 years old living in informal settlements, there are an estimated 32,000 cases of typhoid fever annually. Targeting these children with a typhoid immunization campaign, assuming vaccine efficacy of 60% (estimates of effectiveness range from 50% to 80% depending upon vaccine and setting [40], [41], and 70% vaccination rates (likely achievable in campaigns in densely populated settings), would prevent approximately 13,500 cases/year of typhoid fever in vaccinated children. In addition, indirect protection would likely reduce transmission from children to other household members or other contacts, as was recently demonstrated in a typhoid vaccine effectiveness study in Kolkata [41].

Failure to recognize and act on typhoid burden and potential for its prevention adds another dimension to currently neglected tropical diseases in urban African settings [42] for which safe and effective prevention options are already available, but are not used. As urbanization continues, addressing such neglected conditions will become more critically urgent.
